# MSDC: Aspect-level sentiment analysis model based on multi-scale dual-channel feature fusion

**DOI:** 10.1371/journal.pone.0328839

**Published:** 2025-10-21

**Authors:** Xiaoye Lou, Guangzhong Liu, Yangshuyi Xu

**Affiliations:** College of Information Engineering, Shanghai Maritime University, Shanghai, China; Kitami Institute of Technology, JAPAN

## Abstract

Aspect-level sentiment analysis is a significant task in the field of natural language processing. It can process text in a fine-grained manner to predict the sentiment polarity of a specific aspect word in a sentence. However, existing single-channel models often ignore high-dimensional local feature information in syntactic dependencies, have a single structure, and cannot fully extract text features. At the same time, there are often multiple opinion words with diverse sentiment attitudes in a sentence, so there is a certain amount of noise when processing features, which interferes with the model’s understanding of the sentiment semantics related to aspect terms. To address the problems, this paper proposes an aspect-level sentiment analysis model (MSDC) based on multi-scale dual-channel feature fusion. First, through multi-head gated self-attention channels and graph neural network channels, the model further enhances its understanding of the spatial hierarchical structure of text data and improves the expressiveness of features. Then, we design an adaptive feature fusion mechanism that dynamically adjusts the weight ratio of aspect words to context according to a given aspect. Hence, the task pays more attention to key information. Finally, the data is integrated and processed through a capsule network. The results indicate that our model exhibits superior effectiveness on multiple public datasets, especially when processing fine-grained text sentiment analysis tasks, significantly improving the accuracy and F1 value compared to existing technologies.

## 1 Introduction

In the age of digital communications, the posting of various comments has become a significant method for individuals to communicate their views. In this context, the specific implementation of sentiment analysis has emerged as a prominent research focus. Aspect-level sentiment analysis (ABSA) is a form of natural language processing that focuses on identifying and analyzing opinions, sentiments, attitudes, and emotions expressed on specific aspects or characteristics in text, such as product reviews or posts on social networks [2]. Distinct from document-level and sentence-level sentiment analysis, which generally assign an overall sentiment to a block of text or a complete sentence, ABSA is responsible for assessing the sentiment orientation for every distinct aspect term in a sentence [1]. Aspect-level sentiment analysis represents a more entity-centric and granular challenge within the field of sentiment analysis. The main objective of aspect-level sentiment analysis is to determine the sentiment polarity (positive, negative, or neutral) of aspect words within a given sentence. This level of analysis is particularly useful for understanding the specific opinions related to different aspects of a product, service or topic, providing more nuanced insights than broader sentiment assessments [2]. There are different sentiment words for sentences with multiple aspect words to represent the corresponding sentiment types. Many current techniques frequently misattribute aspect words to incorrect sentiment words, leading to inaccuracies in sentiment analysis. Let’s look at the following sample sentence from the SemEval2014 dataset [24] to illustrate this problem:

“The fish is fresh but the variety of fish is nothing out of ordinary”

**Fig 1:**
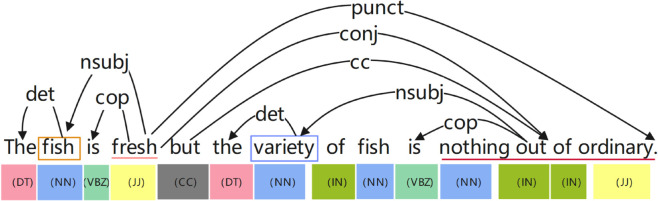
Syntax structure diagram.

The grammatical structure is shown in Fig 1. In this sentence, the aspect words are “fish" and “the variety of fish", and the corresponding modifiers are “fresh” and “nothing out of ordinary". These two aspect terms are expected to have positive and negative sentiment polarity, respectively. The sentiment orientation of aspect terms is vital in applications like product review analysis, because it is necessary to understand users’ opinions on specific aspects of products, which is of particular reference value to enterprises, and to take corresponding measures based on these opinions [3]. However, existing methods will incorrectly combine two aspect words with their adjacent words when performing sentiment analysis, such as matching “variety" with “fresh", resulting in sentiment analysis errors and certain losses to enterprises.

To mitigate the issue of misalignment between aspect and sentiment words in aspect-level sentiment analysis, it is vital to construct a semantic linkage between an aspect term and its related opinion word. A range of recurrent neural networks (RNNs) [4,5] have been suggested for direct learning from the surrounding context of an aspect term. However, with increasingly complex text information, accurately demonstrating how aspect words relate to both short-distance and long-distance situations is difficult. Hence, diverse attention mechanisms are extensively applied in ABSA to address the associated challenges, encapsulating the relationship between aspect terms and their context [6,7]. Unlike RNN-based approaches, attention mechanisms offer robust modeling capabilities, enabling them to capture the remote associations between aspect terms and the surrounding text [8]. However, attention mechanisms do not work well when processing sentences containing multiple aspect words. For example, a given aspect word “fish" can be associated with the opinion words “fresh" and “nothing out of the ordinary" simultaneously. In such scenarios, the attention mechanism might prioritize matching opinion terms to the relevant aspect terms, which can lead to a misalignment of semantics [9]. To investigate the intricate associations between aspect terms and surrounding lexical elements, graph-theoretic techniques leverage network topologies to encapsulate inter-lexical dependencies, subsequently refining the semantic embeddings of individual tokens via the propagation mechanism of Graph Convolutional Network (GCN). With the emerging development of graph machine learning [10–12], and the semantic parsing technology proposed by Xie et al. [14], Zhou et al. [18] presented the RDGCN to enhance the dependency tree weight calculation problem in ABSA by introducing a reinforcement learning mechanism. The accuracy of aspect-level sentiment analysis based on graph neural networks is gradually improving.

Despite their notable effectiveness, the aforementioned techniques nonetheless have certain drawbacks: (1) Lack of relationship: As mentioned above, both the attention mechanism and the graph neural network-based model can capture dependencies for sentiment analysis. However, the relationship between semantic sentences cannot be adequately captured by ordinary dependencies alone. They perform poorly in processing increasingly complex sentences. (2) Lack of interaction: Most benchmark methods only focus on the structured relationship of the data when processing but do not consider the profound relationship between the data. When performing sentiment analysis, they combine the data and do not consider the interactive connection between sentences. (3) Lack of target: The precise relationship between the sentence’s aspect and opinion words cannot be ascertained. When processing text data, the above methods do not fully utilize the relationship between global and local features in the sentence, resulting in some feature information loss during the processing. The impact of text sentiment analysis will be impacted by the noise that is produced during positioning.

The goal is to address the issue of insufficient feature processing in existing models. By enhancing the capture of high-dimensional local features in syntactic dependencies, reducing the noise interference caused by multiple opinion words, it can analyze the sentiment polarity of specific aspect words more accurately. The contributions of this paper include proposing an aspect-level sentiment analysis model based on multi-scale dual-channel feature fusion. We introduce an aspect-level sentiment analysis model that leverages multi-scale dual-channel feature fusion to address the previously discussed issues and shortcomings. Initially, we incorporate a domain enhancement module that applies dynamic masking to the data processed by BERT, thereby accentuating the associations between neighboring words and aspect terms. Then, in order to capture more comprehensive feature information, a dual-channel mechanism is designed in this model to process the features. By introducing a multi-head gated self-attention module, each aspect of the multi-angle sentiment representation of the model is represented by an abstract understanding. Concurrently, features are learned via a graph neural network, which concentrates on capturing the local characteristics of the features. This parallel channel processing enhances the precision and thoroughness of identifying pertinent opinion words related to a specified aspect, and it also boosts the model’s ability to extract more nuanced features. Then, the two extracted features are adaptively fused and processed through the capsule network. Combining the hierarchical feature aggregation ability of the capsule network, it realizes the fusion and precise expression of multi-scale features. While capturing local features, the global semantic structure is accurately modeled to achieve multi-scale fusion and hierarchical expression of features and alleviates the problem of semantic misalignment. Ultimately, the global average pooling layer consolidates data from every contiguous span, yielding a holistic representation of the sentiment conveyed towards a specific aspect. This process effectively addresses the challenges of semantic misalignment and insufficient feature depiction.

The following is a summary of the study’s main contributions:

(1) In the BERT-based model, the matrix of self-attention parameters is initialized and retrained. However, the self-attention weights directly obtained from BERT are ignored, and BERT is pre-trained with large-scale data.

(2) The current paper introduces the Multi-Scale Dual-Channel feature fusion model (MSDC), a novel architecture aimed at addressing the semantic mismatch issue. This approach effectively leverages the contextual relationships of various contiguous intervals, guaranteeing the precise mapping between sentiment expressions and specified aspects. The MSDC model incorporates a domain enhancement module that emphasizes the interaction between adjacent terms and the target aspects, thereby extracting more salient information related to the aspect in question. A dynamic masking strategy is introduced in the BERT encoding layer. By adaptively masking the non-critical context, the semantic association between neighboring words and the target aspect words is strengthened, providing high-quality input for subsequent feature extraction. At the same time, the global features obtained by multi-head gated self-attention and the local features obtained by the enhanced graph neural network are fully fused through the adaptive feature fusion module. Lastly, the capsule network is utilized to further extract the fused feature information from the data features. The comprehensive opinion information is aligned with the given aspect in a parallel manner, and the relevant information of the aspect words is fully extracted.

(3) Experiments on benchmark datasets such as SemEval-2014 and Twitter have shown that the MSDC model significantly outperforms existing methods in terms of both accuracy and F1 score. It performs particularly outstandingly in sentences with complex syntax or multiple conflicting opinions. The empirical findings indicate that our introduced framework surpasses alternative methods.

This piece of writing is structured as follows: Sect 2 explores prior research, underscoring the scholarly efforts relevant to our investigation; Sect 3 provides a detailed description of the suggested model; Sect 4 reports on the experiments that were carried out and their outcomes; Sect 5 reflects research insights and impact Discussion; Sect 6 wraps up our conclusions and contributions.

## 2 Related works

### 2.1 Aspect-level sentiment analysis

An essential subfield of sentiment analysis research is ABSA. It can anticipate the sentiment polarity of a sentence’s specific feature by processing fine-grained text [20,21]. ABSA finds extensive utility in areas such as evaluating product feedback, analyzing social media content, and conducting market studies. It can help companies understand customer feedback on specific product features or services in detail, thereby achieving more targeted improvements. ABSA’s core function is to accurately identify and analyze the sentiment orientations toward particular elements or entities in a text. ABSA requires that one focus on the aspect and sentiment-specific terms in the text and understand how they interact.

It is essential to take into account both the subtle nuances of sentiment expression and the semantic elements present in the context while doing sentiment analysis. Traditional sentiment analysis approaches assess the overall sentiment direction of the text in its entirety [22,23], whereas ABSA targets the sentiment associated with each aspect within a sentence. This fine-grained approach can evaluate opinions on specific entities. Previously, the primary methods for extracting sentence representations included recurrent neural networks and convolutional neural networks. Nevertheless, due to the architectural constraints of these models, they typically fail to effectively capture and encapsulate the syntactic and semantic features within sentences. The majority of the most recent aspect-level sentiment analysis techniques now in use makes use of graph neural network models. A specific type of deep learning called graph neural networks is made to handle graph data. It uses the syntactic structure of sentences to establish connections between aspect terms and the context in which they are used [15,18].

Meanwhile, in the field of ABSA, the application of capsule networks is gradually on the rise [52,53]. Compared with traditional methods, the capsule network precisely models the association between local and global text semantics through dynamic routing, accurately parsing the interaction between aspect terms and context in ABSA. Vectorized representation and multi-routing characteristics enhance the model’s capability to analyze sentiment semantics and polysemous words [39].

### 2.2 BERT model

Google’s research team proposed the BERT (Bidirectional Encoder Representations from Transformers) [28] model. BERT introduced a bidirectional context pre-training method, markedly enhancing the efficacy in numerous natural language processing challenges. Compared with the two text embedding methods, GloVe and Word2Vec, BERT has better results in context awareness, pre-training and fine-tuning, long-distance dependency processing, multi-task learning, and overall performance [29]. BERT preprocessing includes several essential steps to ensure the model can correctly process the input data. To handle rare words, the input text is first divided into words, and then the WordPiece technique is applied to break the text up into smaller word fragments. Then, BERT adds the “[CLS]” identifier at the beginning of the input to represent the classification information of the entire sequence and adds the “[SEP]” identifier at the end of each sentence to distinguish the boundaries between sentences. Then, the input tokens are converted into the corresponding ID through the vocabulary list, and the corresponding attention mask is generated to distinguish valid words from filler parts. At the same time, BERT generates segment IDs to distinguish different sentences and assist the model in comprehending the connections between phrases. Finally, the input data is padded to a uniform length to ensure input consistency during batch processing.

### 2.3 Graph neural networks

Within ABSA, graph neural networks have demonstrated significant promise. The Graph Convolutional Network (GCN) is a type of deep learning architecture specifically crafted for handling data structured in graph form [17]. The core of GCN is to aggregate a node’s features with those of its adjacent nodes using convolutional operations. This process encapsulates the local structure’s features within the graph, ensuring that a node’s final representation encompasses not only its own information but also incorporates the data from its surrounding nodes. This aggregation allows the model to effectively understand the graph’s overall structure. For example, Zhang et al. [30] pioneered using GCN for ABSA. They proposed implementing GCN on a syntactic dependency tree to effectively utilize aspect word context and dependency information in a sentence. Huang et al. [31] and Sun et al. [32] also proposed ABSA models based on graph neural networks to process features from multiple perspectives. In the ABSA task, graph neural networks are very effective in enhancing the role of context and aspect word dependencies.

The role of Graph Attention Networks (GAT) in ABSA is mainly reflected in its ability to effectively process unstructured data and capture complex dependencies, especially the relationship between words in text data [48]. Studying the sentiment tendency of particular textual elements (such product characteristics) is known as ABSA. This often entails figuring out how each word in the text is related to a specific phrase or component. Text analysis can more precisely capture the association between words and between words and aspect words because to GAT’s special attention mechanism, which allows it to give distinct weights to each edge in graph-structured data [49].

### 2.4 Multi-head self-attention mechanism

The multi-head self-attention mechanism plays a significant role in sentiment analysis [33]. It meticulously captures multi-level emotional and semantic information in text by processing multiple attention heads in parallel. To have an accurate understanding of the text’s emotion, each attention head can concentrate on distinct textual elements and different kinds of interactions, including grammatical structures, contextual impacts, and word emotional tendencies. This mechanism enables the model to identify subtle emotional differences in a complex text, such as sarcasm, puns, or implicit expressions, improving the accuracy and depth of sentiment analysis. In specific applications, multi-head self-attention can effectively process diverse texts such as user comments, social media posts, or customer feedback and provide enterprises with more accurate customer sentiment analysis and market trend predictions by capturing profound semantic and emotional clues [2]. This efficient information processing capability makes the multi-head self-attention mechanism an essential technology in sentiment analysis, significantly improving the overall performance of natural language processing systems.

### 2.5 Capsule networks

The capsule network was introduced by Hinton et al. [34], addressing the flaws and limitations of RNN and CNN, and has yielded promising outcomes in text categorization tasks. A collection of neurons that encodes a certain thing or item makes up a capsule. By sending vector-based data to the layer below, each capsule exchanges information with the capsules in the layer above. The term “dynamic routing" refers to this approach. In ABSA, the capsule network enhances the understanding of the context of the sentence and its internal structure by capturing the spatial relationship and syntactic structure between words. Compared with traditional convolutional neural networks, the capsule network can better preserve the hierarchical relationship and dependency between words and is thus more accurate in identifying aspect-related sentiment features. Its dynamic routing mechanism also improves the capture and classification of sentiment information in multi-aspect sentences, making it perform well in complex sentiment analysis tasks.

## 3 Methods

**Fig 2:**
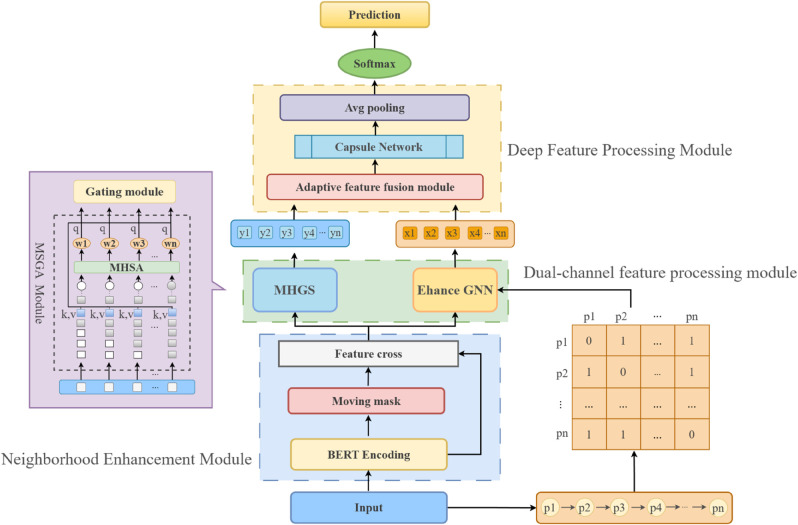
MSDC model structure.

Using the Multi-Scale Dual-Channel feature fusion (MSDC) method suggested in this work, this chapter will explore the aspect-level sentiment analysis strategy. Fig 2 illustrates the MSDC model’s architecture.

The MSDC model is primarily composed of three modules: a feature extraction component, a multi-scale feature handling component, and a feature augmentation component. Sect 3.1 delineates the process by which the model extracts features from comprehensive text reviews. The model’s processes for multi-scale feature processing and feature improvement are then covered in depth in Sects 3.2 and 3.3, respectively.

For the MSDC model’s processing workflow demonstrated in the following example: Input Sentence: “The battery life is impressive, but the camera quality disappoints”. Target Aspect Term: “camera quality”. The MSDC model first establishes the dependency relationship “disappoints – camera quality” through syntactic dependency parsing, enriches deep feature representations via a neighborhood enhancement module, then captures negative sentiment features along syntactic paths using a Graph Neural Network. Simultaneously, it strengthens semantic correlations between the aspect term and “disappoints” through a multi-head attention mechanism. Subsequently, an adaptive fusion mechanism dynamically integrates dual-channel features while filtering irrelevant noise like “battery life”. Finally, the capsule network routes fused features to the negative sentiment polarity, ultimately outputting a negative sentiment determination for “camera quality”.

### 3.1 Feature extraction module

#### 3.1.1 BERT pre-training.

**Fig 3:**
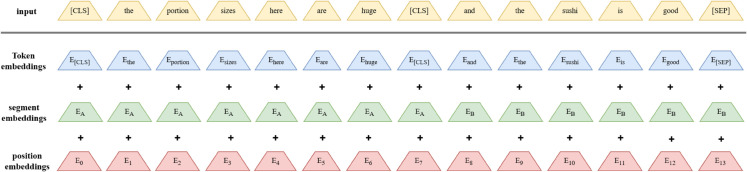
BERT preprocessing.

To achieve a more comprehensive contextual encoding of sentences, we utilize a pre-optimized BERT architecture that converts input sentences into vector formats $H\in R^{n+1\times d}$, where n represents the length of the sentence and d represents the embedding dimension. The representation of this model contains a unique tag [CLS] for classification and n word tags, thus providing the global semantics of the sentence and the local representation of each word. To further capture the aspect-level and contextual sentiment analysis of sentences [35,36], we construct an input pair that combines sentences and specific aspects in the form of “[CLS] sentence [SEP] aspect word [SEP]”, which is used as the input of the BERT. This architectural approach not only preserves the semantic content of the sentence but also facilitates the model’s focus on the context surrounding individual aspect terms. Through the explicit representation of the linkage between sentences and aspect terms, the model’s proficiency in comprehending specific tasks is enhanced. After encoding, BERT generates an aspect-aware contextual representation $H=\{h_{CLS},h_{1},h_{2},\ldots ,h_{n}\}$, where $h_{CLS}$ is the global sentence representation and $h_{1},h_{2},\ldots ,h_{n}$ represents the embedding of each word in a specific context. This representation sequence is then input into subsequent modules, allowing our model to more effectively capture the deep semantic relationships of sentences and the fine-grained semantic differences of specific aspects, thereby improving performance in the following steps. The processing form is shown in Fig 3.

#### 3.1.2 Neighborhood enhancement module.

Inspired by the AOAN model [27], this paper builds multiple neighboring word combinations to highlight different ranges of neighboring word combinations related to a given aspect, thereby enhancing the model’s capture of the association between aspects and context. Considering that words surrounding a certain aspect word frequently include information useful for predicting emotion polarity [37,38], we construct several adjacent spans to represent the tokens surrounding the aspect term, encompassing the aspect term. The adjacent spans encompass diverse ranges around the aspect, each confined to the particular positional scope.

Specifically, we initially employ the moving mask mechanism to determine the relative location of each token relative to the aspect word referenced from [27]. Subsequently, we compute the relative distance *d*_*j*_ for each token in relation to the aspect word, defined as follows:

$$d_{j}=\left\{ \begin{array}{cc} M_{a+1}-M_{j} & j<a+1\\ 0 & a+1\leq j\leq a+m\\ M_{j}-(M_{a+1}+m) & j>a+m \end{array}\right.$$ (1)

In the above formula, the variable *M*_*j*_ denotes the position of the number of j token, with j varying from 0 to n, where n represents the input sentence’s length. *M*_*a* + 1_ represents the aspect’s starting position, while m indicates its length. To represent the varied contexts of bordering words, then create multiple spans based on the threshold size T, ranging from 0 to T. This method enables us to generate T+1 neighboring spans. The mask vector $V_{j}^{t}$ for every surrounding term in the range t∈[0,T] is defined as follows:

$$V_{j}^{t}=\left\{ \begin{array}{cc} E & d_{j}\leq t\\ O & d_{j}>t \end{array}\right.$$ (2)

$V_{j}^{t}$ represents the masking vector for j items with segment length t, where $E\in \;R^{d}$ is a vector and $O\in \;R^{d}$ is a null vector. The following equation provides the contiguous segment representation ${H^{t}}_{span}\in \;R^{(n+1)\times d}$, where t is the segment’s length:

$$H_{span}^{t}=M^{t}\cdot H$$ (3)

In which the masking matrix $M^{t}=\left\{V_{0}^{t},V_{1}^{t}...V_{n}^{t}\right\}\in R^{(n+1)\times d}$ is composed of n+1 masking vectors $V_{j}^{t}$, each denoting the segment of length t from the input sentence.

Concatenating the adjacent span representation with the original context representation H yields the adjacent span enhancement representation ${H^{t}}_{enhance}\in \;R^{(n+1)\times d}$. A common linear transformation is then used to project these vectors into a single d-dimensional semantic space, which is represented as follows:

$$H_{enhance}^{t}=W_{1}\left(H_{span}^{t}⊙H\right)+b_{1}$$ (4)

where ⊙ stands for the cascade operation and $W_{1}\in R^{d\times 2d}$ and $b_{1}\in R^{d}$ are the linear transformation layer’s weight and bias matrices.

### 3.2 Multi-scale feature processing module

Inspired by the dual-channel model [19,35], this model features a dual-channel processing architecture that includes a multi-head gated self-attention module and an advanced graph neural network module. These two channels process input data from different perspectives. Specifically, the multi-head gated attention mechanism is used to capture the global dependency of words in a sentence, ensuring that each word can obtain dynamic context information based on other words in the sentence. At the same time, the graph neural network channel utilizes graph convolution and attention mechanisms to examine adjacency relationships in the input text, extracting local structural features of neighboring words. In graph convolution, the adjacency matrix is used to aggregate text nodes to capture the local context information in the sentence, and the weights of different nodes are adaptively assigned through the graph attention mechanism to ensure that important neighbor information is better represented. Finally, the feature information of the previous two layers is integrated through the graph convolution layer.

The advantage of this architecture is its flexible dual-channel design, which can not only capture the sentence’s overall semantic structure but also consider the local relationship between adjacent words. By combining the attention mechanism and graph neural network, the model can improve its understanding and modeling capabilities of semantics at different levels of granularity.

#### 3.2.1 Multi-head gated self-attention module.

(1) Multi-Head Self-Attention Mechanism (MHSA)

To improve the representational ability of distinct modality features, the encoding and decoding portions employ a multi-head self-attention mechanism. The query matrix Q, the key-value matrix K, and the value matrix V are first restructured into H matching sub-query matrices, sub-key-value matrices, and sub-value matrices, all of which have the same dimensions. The following formula describes the reconfiguration in detail:

$$Q=XW_{Q},K=XW_{K},V=XW_{V}$$ (5)

Where X is the input vector, $W_{Q},W_{K},W_{V}$ are trainable weight matrices.

The self-attention mechanism maps each vector (embedding of a word) of the input sequence into a query (Query, Q), a key (Key, K), and a value (Value, V) matrix. The formula is as follows:

$$MultiHead(Q,K,V)=Concat({head}_{1},{head}_{2},...,{head}_{h})W_{O}$$ (6)

Where $W_{O}$ is the output weight matrix and h is the number of heads. Concat() represents the operation of connecting all heads. The multi-head attention mechanism enables the model to capture contextual dependencies across various subspaces.

The mechanism then conducts attention computations individually across each head. Afterward, the results from all heads are concatenated together to form the ultimate feature representation. The computational procedure is detailed below:

$${head}_{i}=Attention\left({QW}_{i}^{Q},{KW}_{i}^{K},{VW}_{i}^{V}\right)=Softmax\left(\frac{{QW}_{i}^{Q}({KW}_{i}^{K}{)}^{T}}{\sqrt{d}}\right){VW}_{i}^{V}$$ (7)

$W^{Q_{i}}\;\in \;R^{d\times dh}\;,\;W^{K_{i}}\in \;R^{d\times dh}\;,W^{V_{i}}\;\in \;R^{d\times dh}$ represents the projection matrix of the number of i head, and $F\in R^{n\times d}$ represents the output features.

(2) Multi-head gated self-attention mechanism (MSAG)

By determining the reciprocal link between input sequences, MHSA is able to capture the sentences’ global semantic properties. However, there is a chance that MHSA might unintentionally draw attention to unimportant parts of the input because it can only calculate attention within a specific range. This can potentially lead to less accurate representations of the data, especially in tasks where it is crucial to focus on specific aspects or contexts within the input sequence. Inspired by the model of Xu et al. [41], this model introduces a multi-head gated self-attention mechanism (MSAG) to solve the abovementioned problems. The feature matrix X is introduced into the MSAG (Multi-Head Self-Attention with Gating) module, where the pivotal characteristics are discerned via the Self-Attention process. Following this, a gating mechanism is employed to extract a more decisive and nuanced set of textual features. The MSAG module is structured around three key sub-layers: it accepts X as the input feature set and models the intra-modal interactions among the words within the text via a multi-head attention mechanism and a subsequent dense feedforward neural network layer.

$$X_{2}=\max(0,X_{1}W_{1}+b_{1})W_{2}+b_{2}$$ (8)

In the present scenario, weight coefficients are represented by $W_{1},W_{2}$ and bias variables by $b_{1},b_{2}$. The gating mechanism layer is used to get the issue feature matrix $X^{\left(L\right)}$ once the feature matrix $X_{2}$ has been obtained. The following is the processing procedure:

$$X_{3}=Concat(X_{2},X_{1})$$ (9)

In this context, $W_{1},W_{2}$ stand for the weight matrices and $b_{1},b_{2}$ for the bias terms. Once the feature matrix $X_{2}$ is obtained, the gated mechanism layer is utilized to produce the issue feature matrix $X^{\left(L\right)}$. The process is described as such:

$$X_{t}=\tanh(X_{3})$$ (10)

$$X_{s}=sigmoid(X_{3})$$ (11)

$$X^{\left(L\right)}=X_{1}\;*\;X_{t}+(1-X_{s})\;*\;X_{2}$$ (12)

**Fig 4:**
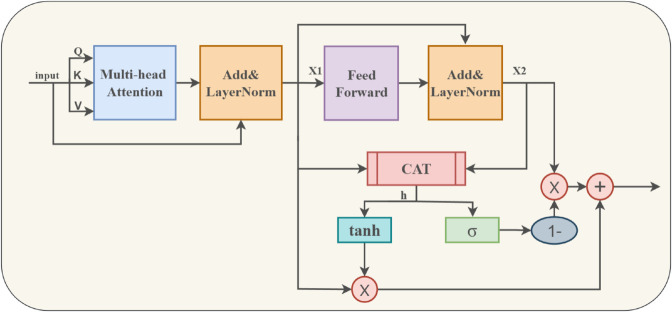
Multi-head gated self-attention mechanism.

Where $X^{\left(L\right)}$ denotes the refined feature matrix ultimately produced by the MSAG unit, having suppressed the extraneous information yielded by L layers of self-attention mechanisms. From Eq (9), the feedforward neural network’s output $X_{2}$ and the MHSA’s output $X_{1}$ can be combined. The integration unifies the semantic relationships revealed by the MHSA with the local textual characteristics harvested by the feedforward network; By utilizing Eqs (10) and (11), the sequential feature vector $X_{3}$ can be successfully confined, enabling the gated linear unit in Eq (12) to dynamically regulate the weight interplay within the feature vector; Following this process, both lower-level and higher-level semantic features are preserved in full, while noise and redundant information are concurrently minimized. The structure can be seen in Fig 4.

#### 3.2.2 Graph neural network module.

(1) Graph Node Representation

Generate dependency graphs (adjacency matrices) for text data to obtain graph-structured relationships of features in aspect-level sentiment analysis tasks [40]. Parse the sentence through spaCy’s dependency parser, converting each word in the sentence and its grammatical dependencies into an adjacency matrix, where every row and column in the matrix represent a sentence word and a value of 1 represents the difference between the two words. There is a grammatical dependency between them, and zero means no dependency. The created dependency graph maintains the identification of words with themselves(the diagonal value is 1) while capturing the dependencies between words. The dependency graph can greatly enhance the model’s capacity to capture complex dependencies between words when processing emotional information related to aspect words. The Eq (13) referenced from [40] shows the construction method.

$$Ti=\left\{ \begin{array}{cc} 1, & \text{ if word }i\text{ depends on word }j\text{ or vice versa}\\ 0, & \;otherwise \end{array}\right.$$ (13)

In this way, additional structured data for aspect sentiment analysis is provided by the dependency graph, which aids in the model’s comprehension of the text’s grammar and context and enhances its capacity to precisely represent sentiment polarity (such as positive, negative, and neutral) at the aspect level. Using the graph construction method, the initial feature matrix can contain rich features related to position information and text semantics. This method is particularly suitable for processing long or complex sentences and this can successfully raise the model’s sentiment analysis task performance.

(2) Graph Convolutional Layer Module

Graph Convolutional Layer: Graph Convolutional Layer: In a graph, G = (V, E), where V stands for the graph’s vertices (or nodes) and E for the edges that link them. In the context of GCN, the aggregation process involves applying convolutional operations to the feature data associated with the graph G. The characteristics of the neighbor nodes that are linked to any particular node in graph G are combined by this convolution process.By incorporating the structural information present in the network and maintaining feature invariance with regard to the relative locations of the nearby nodes, this approach makes it easier to update a node’s features. Consequently, each node in the graph can comprehensively learn not only its own high-level attributes but also the complex interdependencies with its higher-order neighboring nodes referenced from [40].

$$X^{\left(l+1\right)}=\sigma \left(W^{\left(l\right)}{\tilde{D}}^{-\frac{1}{2}}\tilde{A}{\tilde{D}}^{-\frac{1}{2}}X^{\left(l\right)}\right)$$ (14)

In the Eq (14), the node feature matrix for the number of l+1 layers is represented by $X^{\left(l+1\right)}$, the node feature matrix for the number of l layers by $X^{\left(l\right)}$, and the weight matrix for the number of l layers is recorded as $W^{\left(1\right)}\;\in \;R^{n\times d}$. The node characteristics are then subjected to linear transformation; Sigmoid() represents a nonlinear activation function; $\tilde{A}\in \;R^{n\times n}$ represents the adjacency matrix A plus the identity matrix I (representing the self-loop of the node), the graph adjacency matrix is denoted by A, the identity matrix by I, and the symmetric matrix of $\tilde{A}$ by D.

(3) Graph Attention Layer Module

Graph Attention Layer: The graph attention layer incorporates an attention mechanism that assigns varying weights to neighboring nodes in the graph [13]. Initially, the node features are transformed into a new feature space via a linear mapping. Subsequently, attention coefficients for each node pair are computed. To stabilize the representation of nodes using self-attention, a multi-head attention mechanism [38] is employed to enhance the model’s representational capacity. In detail, the K-head independent attention processes are carried out according to the respective equations, and the outcomes of these attention mechanisms are averaged to obtain the final representation. For each node $e_{i}$ and its neighbor node $e_{j}$, the attention coefficient $e_{ij}$ between them is calculated, and the neighbor attention coefficient of each node is normalized using the softmax function. Finally, the neighbor node features are weighted and aggregated, and the normalized attention coefficient $a_{ij}$ is used to weight the features of the neighbor nodes. The representation of the updated node hi is shown in the following equations : where C is the weight matrix in the number of D attention mechanisms, and A is the normalized attention value based on the number of B attention mechanisms.

$$e_{ij}=LeakyReLU\left({\vec{a}}^{𝖳}[W\cdot h_{i}||W\cdot h_{j}]\right)$$ (15)

$$\alpha_{ij}=\frac{\exp(e_{ij})}{\sum_{k\in 𝒩(i)}\exp(e_{ik})}$$ (16)

$${\vec{h^{\prime }}}_{i}=\sigma (\frac{1}{K}\sum_{k=1}^{K}\sum_{j\in N_{i}}W^{k}\alpha_{ij}^{k}{\vec{h^{\prime }}}_{j})$$ (17)

where $W^{k}$ is the weight matrix in the amount of k attention mechanisms, and $\alpha_{ij}^{k}$ is the normalized attention value based on the number of k attention mechanisms.

(4) Graph Neural Layer Combination Module

**Fig 5:**
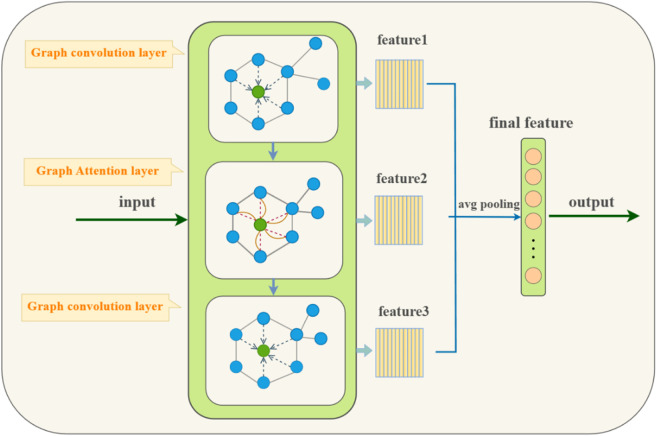
Graph neural network architecture.

Drawing inspiration from the GCNGAT model [26], we have developed an advanced graph neural network. To ensure a comprehensive information extraction method, this improved module combines the graph convolutional layer with the graph attention layer. As depicted in Fig 5, two graph convolutional layers and one graph attention layer make up the structure of the graph neural network module, which is tasked with identifying the relationships between the elements of the adjacency matrix of the input data. The module features a sequential relationship between layers, where the output from the first graph convolutional layer serves as the input to the first graph attention layer, and the output from this attention layer then feeds into the second graph convolutional layer. This progression is illustrated in Eqs (18), (19), and (20) referenced from [26]. The ultimate output graph constitutes the feature vector of the learning module, which is synthesized from the feature vectors acquired at each layer.

The global average pooling layer plays a critical role in the neural network architecture by consolidating the various sets of features extracted from the nodes and effectively reducing the dimensionality of the node features to a uniform size. As indicated in Eq (21), after passing through the global average pooling layer, the final feature representation is achieved.The graph attention mechanism is instrumental in dynamically adjusting the inter-node weights, allowing the model to focus on more relevant connections within the graph. Subsequently, the feature information from neighboring nodes is further disseminated and aggregated via the graph convolution layer. The model iteratively refines the node representations through two successive graph convolution operations, thereby enhancing the precision of feature extraction. The architecture is designed to learn from local feature representations by recursively stacking graph convolution and graph attention layers. This stacking process allows the model to delve deeper into the data, capturing nuanced variations in complex sentiment expressions and providing a comprehensive understanding of the emotional landscape within the text. The ultimate output from the graph representation learning component is a merge of feature vectors derived from each stratum in sequence. This amalgamated feature vector empowers the model to more adeptly detect nuanced variations in sentiment, thus providing a more profound and intricate understanding of the affective architecture inherent within the text.

$$H^{\left(1\right)}={\mathrm{GCN}}_{1}(G,X)=\mathrm{ReLU}\left(W_{0}X{\tilde{D}}^{-\frac{1}{2}}(G+I){\tilde{D}}^{-\frac{1}{2}}\right)$$ (18)

$$H^{\left(2\right)}={GAT}_{1}\left(G,H^{\left(1\right)}\right)=ReLU\left(\frac{1}{K}\sum_{k=1}^{K}\sum_{j\in N_{i}}W_{1}^{k}\alpha_{ij}^{k}H_{j}^{1}\right)$$ (19)

$$H^{\left(3\right)}={GCN}_{2}\left(G,H^{\left(2\right)}\right)=ReLU\left(W_{2}H^{\left(2\right)}{\tilde{D}}^{-\frac{1}{2}}(G+I){\tilde{D}}^{-\frac{1}{2}}\right)$$ (20)

$$H^{\prime }=\text{average pool}(H^{\left(3\right)}∥H^{\left(2\right)}∥H^{\left(1\right)})$$ (21)

G and X serve as the connectivity matrix and attribute features of a particular subgraph within the text vector, respectively. H’ constitutes the ultimate feature depiction.

GCN can effectively capture the local structural information between nodes through graph convolution operations. It can learn the structural relationship between words and their context. GAT can dynamically adjust the weights of the edges between different nodes through its attention mechanism, which means that the influence of particular words can be automatically strengthened or weakened according to the importance in the context. In aspect-level sentiment analysis, combining these two networks means that the model can understand the fundamental structural relationship between words and dynamically adjust the importance of these relationships for specific aspects. For example, for the sentence “The service in this restaurant is good, but the price is on the high side", the model needs to distinguish which words are related to “service" and which are related to “price", and assign different weights according to the sentiment tendency in the context. This combination can improve the flexibility and accuracy of the model because it not only relies on fixed structural information but also adjusts the degree of attention to different words according to the needs in the actual context and better processes important information in the local area.

### 3.3 Feature extraction module

#### 3.3.1 Adaptive feature fusion module.

**Fig 6:**
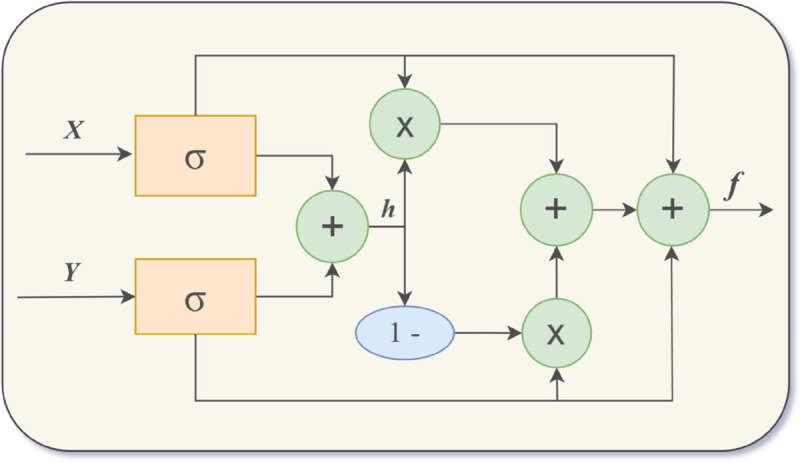
Adaptive feature fusion module.

To enhance the efficacy of feature integration for the ultimate sentiment analysis objective, it’s crucial to refine the text feature vector X and the graph neural network feature vector Y, as they may still retain extraneous noise. Consequently, we introduce an Adaptive Feature Fusion Module (AFFM) to address this challenge, as illustrated in Fig 6, influenced by the scholarly works of Dosovitskiy, Xu et al. [39–42]. The AFFM fine-tunes the output feature data by manipulating two distinct channels, thereby proficiently merging the two feature sets. This strategy enables a more thorough grasp and interpretation of the nuanced emotional and semantic variances within the text. Simultaneously, the integration of features across multiple scales enhances the model’s capacity for generalization, allowing it to absorb and interpret more sophisticated semantic fusion features. The AFFM calculates the dynamic integration of characteristics from the textual feature array X and the graph vertex feature array Y to produce the merged feature vector f. The algorithmic procedure is outlined as follows:

$$h_{t}=\mathrm{sigmoid}(X)$$ (22)

$$h_{g}=\mathrm{sigmoid}(Y)$$ (23)

$$h=(h_{t})+(h_{g})$$ (24)

$$Z_{t}=h\;*\;(h_{t})$$ (25)

$$Z_{g}=(1-h)\;*\;(h_{g})$$ (26)

$$f=Z_{t}+Z_{g}+h_{g}+h_{t}$$ (27)

In the calculation process described, the critical step involves executing a profound and detailed filtering of information on feature X from the multi-head gated self-attention mechanism and feature Y from the graph neural network, along with computing their weighted and transformed feature h via the previously mentioned equations. The equations can be used to dynamically adjust $h_{t}$ and $h_{g}$, the global adaptive feature $Z_{t}$ and the local adaptive feature $Z_{g}$, to adaptively control the inflow of feature information and realize the integration of global feature representation and local feature representation. The AFFM produces a combined feature depiction f and subsequently conducts additional feature manipulation.

#### 3.3.2 Capsule network module.

**Fig 7:**
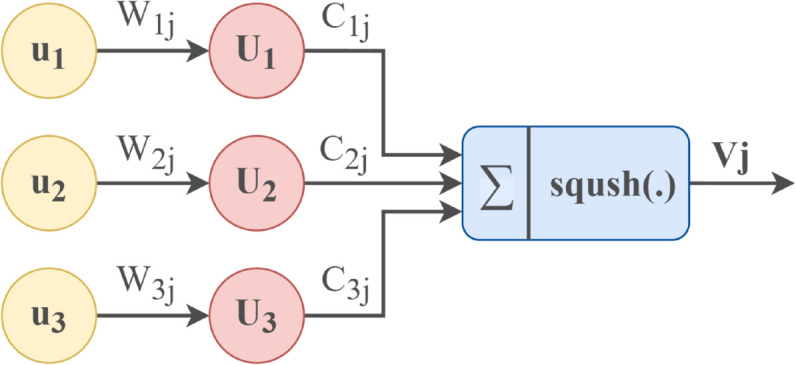
Schematic diagram of the internal principle of the capsule.

The capsule network can further capture the spatial relationship and hierarchical structure in the fused features of the two channels through multi-dimensional representation and dynamic routing, thereby generating a more refined feature representation. The capsule network’s dynamic routing mechanism enables it to process the features after dual-channel fusion selectively. It can adaptively decide which features are helpful for the final task and which features can be ignored. Through this mechanism, CapsNet can focus on extracting the most beneficial information for sentiment analysis tasks from the fused features, further improving the accuracy and robustness of the model. The illustrative plot of the internal mechanism of the capsule is shown in Fig 7 below.

The capsule is located in the number of i layer. The process of generating the prediction vector *u*_*j*|*i*_ according to the input vector $u_{i}$ is shown in Eq (28) referenced from [52,53], where $W_{ij}$ is the weight matrix.

$$u_{j|i}=W_{ij}\cdot u_{i}$$ (28)

**Fig 8:**
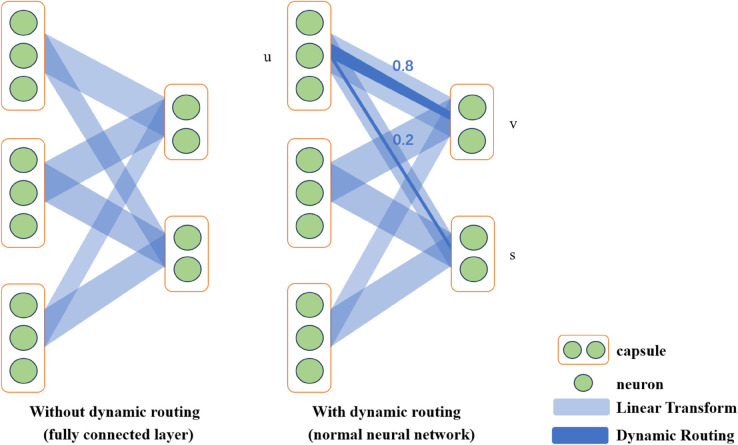
Dynamic routing mechanism.

Dynamic Routing: The dynamic routing mechanism selects which upper-layer capsules the input capsule will pass information to, as shown in Fig 8. Through multiple iterations, the network can determine which upper-layer capsule is more suitable for receiving information from the lower-layer capsule. The network first assigns an initial routing coefficient $b_{ij}$ to each input capsule i and output capsule j, and the initial value is usually set to 0. Routing coefficient update: In each iteration, the routing coefficient $c_{ij}$ is updated so that the capsule associated with the correct pattern contributes more to the transmission of information. The formula referenced from [52,53] is as follows:

$$c_{ij}=\frac{\exp(b_{ij})}{\sum_{k}\exp(b_{ik})}$$ (29)

$b_{ij}$ is the initial connection weight between input capsule i and output capsule j, and $c_{ij}$ is the normalized routing coefficient, which indicates the probability of capsule i being transmitted to capsule j. Through this soft routing mechanism, the capsule network can dynamically adjust the information flow to ensure that important information is transmitted to relevant capsules rather than noise information.

The weighted sum of the primary capsule layer information calculates the output capsule layer referenced from [52,53]. Specifically, the output capsule $s_{j}$ is the weighted sum of all its input capsules, and finally, the output vector $v_{j}$ of the capsule layer is obtained after nonlinear transformation by the nonlinear activation function Squash:

$$s_{j}=\sum_{i}c_{ij}u_{j|i}$$ (30)

$$v_{j}=\frac{∥s_{j}{∥}^{2}}{1+∥s_{j}}\frac{s_{j}}{∥s_{j}∥}$$ (31)

The image neural channel in the dual channel is responsible for capturing local contextual dependencies, and the other multi-head gated self-attention channel is responsible for capturing global features. The capsule network can further build a more hierarchical representation based on the fusion. The capsule network processes the fused features through its squash function and dynamic routing mechanism. CapsNet can filter out possible noise, especially in the scenario of multi-source information fusion, and retain the most compelling features, thereby enhancing the accuracy of sentiment classification. The capsule network can generate more accurate sentiment predictions for each aspect word when processing these fused features.

### 3.4 Sentiment classification

The combined representation $\hat{v}$ can be used in the softmax function to predict the distribution of sentiment polarities:

$$\hat{v}=Softmax(W_{o}v_{c}+b_{o})$$ (32)

where $\hat{v}\in R^{3}$ denotes the anticipated sentiment orientation, $b_{o}\in R^{3}$ and $W_{o}\in R^{3\times 3}$ are the trainable parameters. The following procedure explains the loss function in order to validate the whole network:

$$L(\hat{v},v)=-\sum_{i=1}^{N}\sum_{j=1}^{C}v_{i}^{j}\log({\hat{v}}_{i}^{j})+\lambda \left(\sum_{\theta \in \Theta }\theta^{2}\right)$$ (33)

Where $v_{i}^{j}$ is the true sentiment classification, C is the count of classifications, ${\hat{v}}_{i}^{j}$ represents the predicted classification, θ denotes each element of the parameter set that is subject to regularization, the parameter for the $L_{2}$ regularization term is denoted by λ, whereas the set of all parameters is represented by θ.

## 4 Experiments

### 4.1 Experimental dataset

For empirical evaluation, we have performed experiments on three established and publicly accessible benchmark datasets. The datasets encompass reviews from the Lap14 (SemEval2014 [24]), Rest14 (SemEval2014 [24]). Furthermore, we include a dataset derived from Twitter(ACL14 Twitter [25]). The Twitter dataset exhibits a skewed class distribution. While we retain the original data distribution to reflect real-world scenarios, our design inherently mitigates imbalance through: Macro-F1 Focus: Prioritizing per-class equality over overall accuracy. Capsule Network Dynamics: Routing-by-agreement naturally suppresses majority-class dominance by requiring consensus among low-level features. Adaptive Attention: The gating mechanism downweights frequent neutral patterns unrelated to target aspects. Each of these datasets is comprised of entries that express one of three sentiment orientations: favorable, impartial, or unfavorable. Moreover, every sentence within these datasets is annotated with the pertinent aspect and its associated sentiment orientation. The details and distribution of the data across these three datasets are summarized in Table 1.

**Table 1:** Dataset distribution.

Data	Positive(+1)	Neutral(0)	Negative(-1)
Restaurant(train)	2164	637	807
Restaurant(test)	728	196	196
Laptop(train)	994	464	870
Laptop(test)	341	169	128
Twitter(train)	1561	3127	1560
Twitter(test)	173	346	173

### 4.2 Experimental parameters and evaluation indicators

The numerical experiments are carried out in the environment of Windows 10 (GPU/RTX 4060Ti, VRAM/16GB, RAM/32GB, Python 3.9). In the experiment, we refer to the recommendations of the literature [18,27] and set the hyperparameters of MSDC as follows: BERT-base-uncased, 768-dimensional word embedding is used as input; The hidden size is set at 768; The Adam optimizer with a learning rate lr of $2\;\times \;10^{-5}$ is used, which selected from the set value of the learning rate in the “4.6 Sensitivity analysis". This learning rate enables the model to converge stably. In the adaptive adjustment mechanism of Adam, it can make the gradient update stable. It can not only avoid slow training but also prevent the disruption of convergence. Moreover, it can reduce overfitting to enhance the generalization ability. Additionally, this learning rate may interact synergistically with other hyperparameters to achieve a good balance. According to the sensitivity of multi-module collaboration, we set the testing range of the dropout value to be between 0.2 and 0.5. When it is uniformly set to 0.3 after comprehensively comparing the performance of all modules, the comprehensive performance is the best. Meanwhile, the F1 score in verification is the highest, and the gap in overfitting is the smallest. To solve the overfitting problem, the model uses $L_{2}$ regularization, in light of the diverse and specific demands for harmonizing components like BERT, GNN, and capsule networks, and in order to construct a mutually reinforcing regularization approach in concert with Dropout and Adam, the regularization weight $\lambda_{2}$ is set to $1\;\times \;10^{-5}$, this value demonstrated the best performance during the experiment; The multi-head gated self-attention mechanism has a fixed number of attention heads of 12, the selection of its value has been specifically analyzed in the “4.6 Sensitivity analysis". The parameters of all models are initialized using a uniform distribution. In consideration of striking a balance between computational efficiency and memory constraints, curbing overfitting, boosting generalization, and suiting the characteristics of capsule networks and graph neural networks, the batch size is configured as 16 in our experiment. The network is trained across 20 epochs, and the maximum sequence length that may be used is 100 tokens.

This study uses accuracy A and macro F1 value as model assessment indicators to assess the effectiveness and performance of the MSDC model. The defining formulas are as follows:

$$A=\frac{T_{TP}+T_{TN}}{T_{TP}+T_{TN}+T_{FP}+T_{FN}}$$ (34)

$$F1=\frac{1}{\left|C\right|}\sum_{i}^{c}\frac{2\times P_{i}\times R_{i}}{P_{i}+R_{i}}$$ (35)

$$P_{i}=\frac{T_{TP}}{T_{TP}+T_{FP}}$$ (36)

$$R_{i}=\frac{T_{TP}}{T_{TP}+T_{FN}}$$ (37)

### 4.3 Comparison of models and results analysis

The proposed MSDC model is contrasted with a number of baseline and advanced models to confirm its efficacy, particularly:

1) ATAE-LSTM [43] uses LSTM enhanced with an attention mechanism, which concentrates on crucial segments related to particular aspects within a sentence.

2) MGAN [46] applies GCN to the original framework of the dependency tree to integrate syntactic data.

3) RAM [45] utilizes a cyclical attention mechanism over sentence memory to highlight the significance of particular aspects.

4) ASGCN [47] employs GCN on the initial structure of the dependency tree to incorporate grammatical details.

5) BiGCN [44] uses a bidirectional propagation structure GCN detection model to perform in-depth analysis of features.

6) R-GAT [50] converts the dependency tree into a relational graph attention architecture in which the connections are delineated by the shortest path and the nature of the dependency, utilizing GAT for amalgamation through an attention-based process.

7) T-GCN [51] differentiates between relation types using an attention mechanism and incorporates attention layer integration to extract features from various GCN layers.

8) DualGCN [35] merges syntactic and semantic data using the Syntactic GCN and Semantic GCN modules simultaneously.

9) SSEGCN [36] captures semantic information via an attention mechanism and augments it with syntactic information by utilizing the minimum tree distance.

10) AOAN [27] captures sentiment information associated with specific aspects through the neighborhood expansion module and multi-view attention mechanism.

11) RDGCN [18] proposes a reinforced dependency GCN, which boosts ABSA through the refinement of grammatical dependency relations by employing diverse tactics.

### 4.4 Experimental results analysis

The information presented in the table is derived from the findings published in the original study. Table 2 reveals that the Accuracy (A) and F1 metrics of the introduced MSDC model have seen a noticeable uplift across the three datasets in comparison to other models. The comparison benchmark models in the experiment include non-BERT models (ATAE-LSTM, MGAN, RAM, ASGCN, BiGCN) and BERT models (AOAN, R-GAT, T-GAT, DualGCN, SSEGCN, RDGCN). This advancement is due to the model’s thorough accounting for the sentiment-related characteristics arising from the aspect interactions within sentences. By merging local and global feature information, the model effectively tackles the challenge of semantic matching inaccuracies, thereby facilitating the extraction of essential features that play a critical role in sentiment analysis.

**Table 2:** Performance comparison experimental results of MSDC model and SOTA methods(missing data is indicated by “-").

Data	Restaurant		Laptop		Twitter	
Indicators	A	F1	A	F1	A	F1
ATAE-LSTM	77.20	-	68.70	-	-	-
MGAN	81.25	71.94	75.39	72.47	72.54	70.81
RAM	80.23	70.80	74.49	71.35	69.36	67.30
ASGCN	80.77	72.02	75.55	71.05	72.15	70.40
BiGCN	81.97	73.48	74.59	71.84	74.16	73.35
**MSDC**	**84.31**	**78.93**	**79.11**	**75.26**	**76.01**	**74.36**
AOAN+BERT	86.43	81.19	82.45	79.09	77.46	76.54
R-GAT+BERT	86.60	81.35	78.21	74.07	76.15	74.88
T-GAT+BERT	86.16	79.95	80.88	77.03	76.45	75.25
DualGCN+BERT	87.13	81.16	81.80	78.10	77.40	76.02
SSEGCN+BERT	87.31	81.09	81.01	77.69	77.40	76.02
RDGCN+BERT	87.49	81.16	82.12	78.34	78.29	77.14
**MSDC+BERT**	**87.83**	**81.76**	**82.41**	**79.38**	**79.21**	**77.24**

Among the above SOTA methods, the primary factor contributing to the suboptimal performance of ASGCN is its exclusive focus on the interactional features between aspects and their contexts, neglecting the interactional features among aspects themselves. As for BiGCN’s underperformance, it might stem from its greater proficiency in capturing English collocations, which makes it less adept at handling expressions with weaker grammatical constructs. In addition, models that simultaneously utilize syntactic and semantic knowledge (DualGCN, SSEGCN) are generally better than models that rely on only one of the methods (R-GAT, T-GCN), which highlights the importance of jointly enhancing syntactic and semantic dependencies. Current standard models that incorporate distance grammars either uniformly decrease distance weights for equidistant elements or obfuscate the initial explicit weights via attention mechanisms and masking, both approaches being less effective than MSDC. In contrast to the DualGCN model, MSDC improves the accuracy of the three datasets by 0.7%, 0.61% and 1.81%; The F1 value increases by 0.60%, 1.28%, and 1.22%, respectively. The experimental results once again prove the effectiveness and generalization of our model. Meanwhile, the introduction of the BERT-based semantic encoding module has significantly improved our model, and the comparison between MSDC and MSDC+BERT can also reveal the role that BERT plays in the operation of the model, reflects that our model has achieved the most significant breakthrough in terms of BERT’s performance, proving that MSDC has acquired more valuable syntactic knowledge for ABSA.

The MSDC model is built upon the BERT-base architecture (110M parameters) and incorporates multi-scale feature fusion modules, including a Graph Neural Network with 1.2M parameters, a multi-head gated attention mechanism with 3.8M parameters, and a capsule network with 4.5M parameters. The total parameter count of the model is 119.5M. The model requires a total training time of 6.7 hours across three standard datasets, with CPU memory consumption ranging from 13.4G to 15.6G during the training process.

### 4.5 Ablation experiment

To explore the function of every modules on the performance of the MSDC.On three datasets, we conducted relevant ablation experiments.

(1) MSDC-NoSpan: The neighboring span enhancement module is removed and the result from the BERT encoding process is applied to the input of the two-channel module.

(2) MSDC-NoMSAG: The MSAG module is removed, and only the GNN retained as the sole channel for feature extraction.

(3) MSDC-NoGNN: The GNN module is removed, and only the MSAG is exploited as the sole channel for feature extraction.

(4) MSDC-NoFuse: Delete the adaptive feature fusion mechanism and only concatenate the features of the GNN module and the MSAG module.

(5) MSDC-NoCapsule: Delete the capsule network module and process the fused features directly.

(6) MSDC-Maxpool: Remove the global average pooling of MSDC and use the maximum pooling to analyse sentiment classification.

The results of the ablation studies are presented in Table 3, the results demonstrate each module’s contribution to the MSDC model’s overall effectiveness. Specifically, the effect of MSDC-NoSpan indicates that the neighborhood enhancement module effectively highlights the importance of neighboring words, thereby improving performance. The comparison of the results of MSDC-NoMHSA and MSDC-NoGNN shows that MHSA enhances the model’s processing of global features; GNN improves the model’s processing of local features. The combined use of the two modules enhances the model’s full utilization of multi-scale features. On the other hand, the effect of MSGA-NoFuse demonstrates the focus of the adaptive fusion module on key information during feature processing. The impact of MSGA-NoCapsule reflects the capsule network’s effective integration of fused features. Furthermore, the experimental performance of MSDC-Maxpool has notably declined, suggesting that equal consideration should be given to each multi-view representation for the final classification stage. In summary, the outcomes of the ablation studies validate the contribution of each MSDC module to enhancing the overall capabilities of the ABSA task.

**Table 3:** Ablation experiment results.

Data	Restaurant		Laptop		Twitter	
Indicators	A	F1	A	F1	A	F1
MSDC-NoSpan	87.70	81.02	82.18	78.16	78.95	76.84
MSDC-NoMSAG	86.61	80.19	81.59	77.81	77.24	77.18
MSDC-NoGNN	87.27	80.84	81.85	77.31	79.26	77.31
MSDC-NoFuse	87.69	81.32	82.01	78.47	78.84	77.11
MSDC-NoCapsule	86.18	81.22	80.55	77.05	78.92	74.40
MSDC-Maxpool	87.51	81.30	82.29	78.93	79.26	76.38
**MSDC**	**87.83**	**81.76**	**82.41**	**79.38**	**79.21**	**77.24**

### 4.6 Sensitivity analysis

In the sensitivity analysis, this study aims to evaluate the model’s sensitivity to different hyperparameters and changes in input features to ensure the robustness and generalization ability of the model.

(1) Learning rate

**Fig 9:**
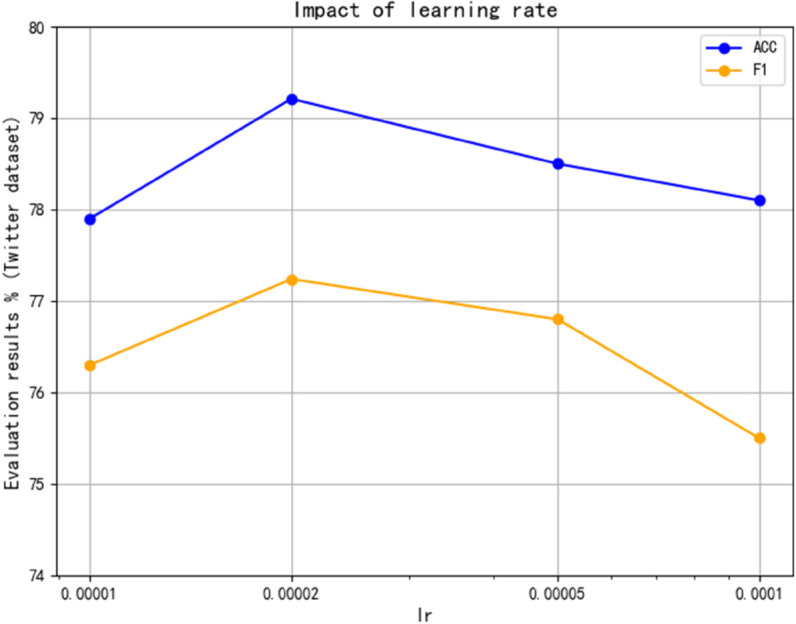
Impact of learning rate on Twitter.

**Fig 10:**
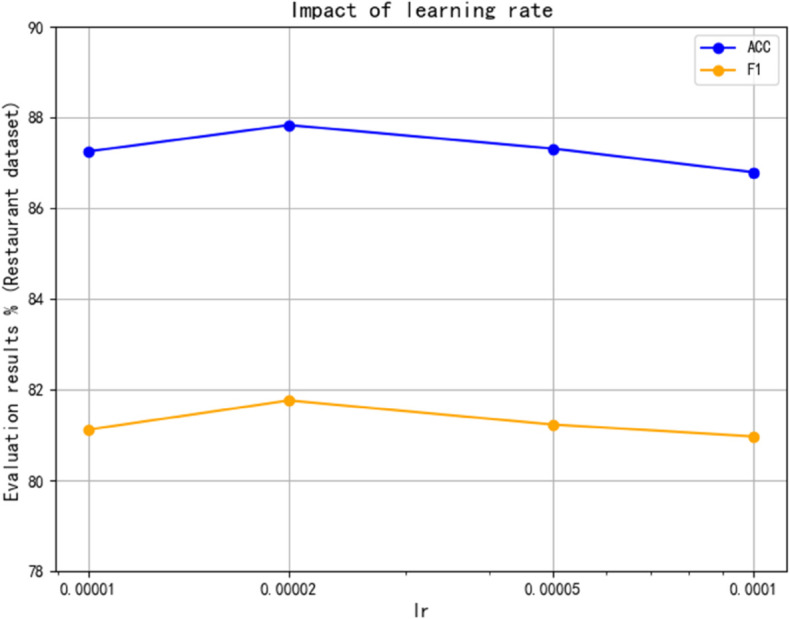
Impact of learning rate on restaurant.

The influence of learning rate on model performance is analyzed. By adjusting the learning rate within a specific range and recording the performance on the dataset, it is found that a learning rate that is too high may cause the loss of function value to oscillate during training, thus affecting the stability of the model. This paper conducts experimental analysis on the Restaurant and Twitter datasets and compares other learning rates. As shown in Figs 9 and 10, an optimal learning rate of $2\;\times \;10^{-5}$ is determined to balance the training speed and convergence quality.

(2) Number of attention heads

**Fig 11:**
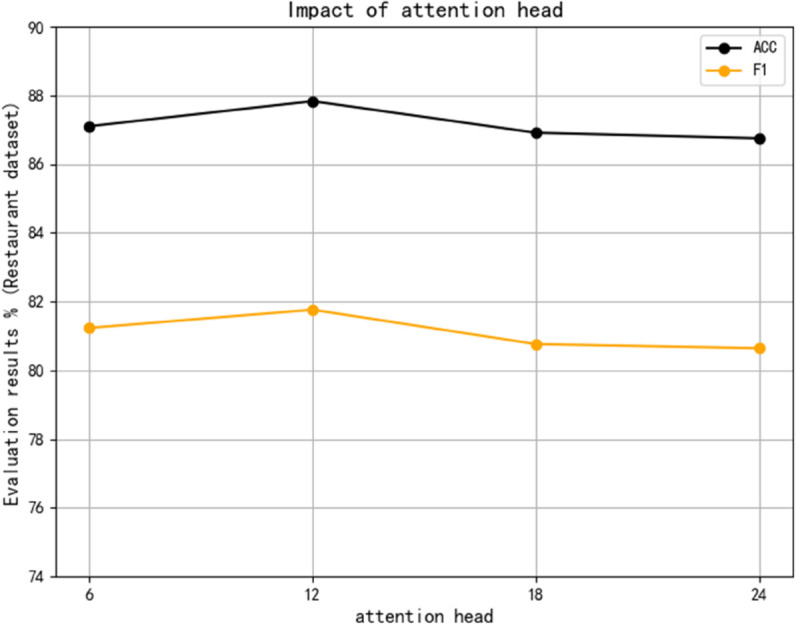
Impact of attention head.

The model presented in this study employs a multi-head gated self-attention mechanism, which enables it to dynamically concentrate on various segments of the input text while handling multiple aspect terms. This mechanism encompasses several attention heads, each of which independently learns to direct its focus on distinct regions of the input to extract information pertinent to different aspects. Each head computes a set of weights that reflect the significance of the input’s various components with respect to a given aspect. Experimental evaluations on the Restaurant dataset were carried out with varying counts of attention heads 6, 12, 18 and 24 to assess their influence on the model’s performance. As shown in Fig 11, the model performs best when the number of multi-head attention heads is set to 12, and the model performs somewhat worse when the number of attention heads is adjusted to other values. The rationale is that a reduced head count restricts the model’s capacity to concentrate on various sizes and locations, raising the risk of data loss. Since the attention head itself is in matrix form, the result is discrete, and there is an error between it and the actual target. If there are more attention heads, the cumulative error may be larger, making the performance of the model worse.

(3) Number of GCN layers

**Fig 12:**
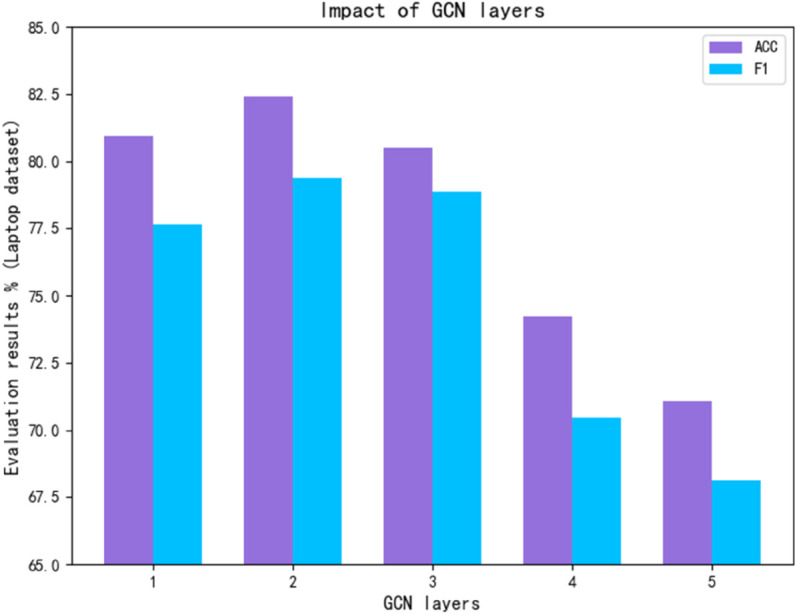
Impact of GCN layers.

The depth of layers in a graph convolutional network is a critical factor influencing model performance. This study performs a sensitivity analysis on the number of GCN layers. The analysis was carried out using the Laptop dataset, with the GCN layer count varied to 1, 2, 3, and 4. The model obtained an F1 value of 77.56% and an accuracy of 81.35% for a single GCN layer. The accuracy and F1 values were 82.41% and 79.38%, respectively, as the number of layers rose to two GCN layers. In comparison to the first layer, the model’s accuracy and F1 value rose by 1.06% and 1.81%, respectively. The model’s capacity to extract syntactic and semantic information was improved. However, it can be seen from Fig 12 that after the second layer was added, the model performance showed a downward trend. For the third layer and more layers, the accuracy and F1 value showed a significant decline. This may be because the model with too shallow layers failed to learn good semantic features, and the network structure was too deep, which caused gradient diffusion or overfitting. Therefore, this paper selected the model with 2 GCN layers for the best effect.

### 4.7 Dataset analysis

The training set of the Twitter dataset contains 6,218 samples, where the neutral class (0) accounts for 49.9%, while the positive (+1) and negative (-1) classes each represent approximately 25%. The test set contains 692 samples, with the neutral class reaching 50.7% and positive/negative classes each around 25%. In contrast, the Restaurant and Laptop datasets exhibit more severe class imbalance. For Restaurant: the training set (3,608 samples) shows a positive class prevalence of 59.8%, neutral class at 17.6%, and negative class at 22.6%, while the test set (1,120 samples) has a dominant positive class (65%) with neutral/negative classes each at 17.5%. For Laptop: the training set (2,328 samples) demonstrates a negative class proportion of 37.4%, neutral class at 19.9%, and positive class at 42.7%, with the test set (638 samples) showing a positive class majority at 53.4%, neutral class at 26.5%, and negative class at 20.1%.

Analysis reveals that Twitter’s neutral-dominated distribution might bias models toward neutral predictions, potentially degrading performance on minority classes and affecting Macro-F1. However, MSDC+BERT achieves a Macro-F1 of 77.24% on Twitter, outperforming counterparts like AOAN+BERT (76.54%) and RDGCN+BERT (77.14%). The minimal performance variance across datasets suggests that class imbalance has limited impact compared to SOTA model capabilities. MSDC’s superior Macro-F1 on Twitter highlights its ability to handle minority classes under neutral dominance, while consistent performance on Restaurant/Laptop demonstrates robust generalization. Experiments confirm that MSDC+BERT maintains stable Macro-F1 across all datasets, including Twitter (77.24%), Restaurant, and Laptop.

In the future, we plan to implement data augmentation strategies, such as AI-driven techniques (e.g., generative adversarial networks or context-aware text generation), to further mitigate the impact of class imbalance. By synthetically expanding underrepresented samples in the neutral, positive, and negative classes, we aim to enhance the model’s ability to generalize across diverse scenarios and improve its robustness in handling minority classes. These augmentations, combined with our MSDC model, are expected to bridge distribution gaps and elevate overall classification performance.

### 4.8 Case analysis

As seen in Table 4, we compare the MSDC with the other three baseline models using a case study on a number of sample words in order to more clearly demonstrate its advantages. The aspect words are included in brackets “[ ]" in each statement. Positive, negative, and neutral sentiments are represented by the capital letters P, N and O, respectively. Every model in the case study uses “BERT-base-uncased" for text encoding in order to provide a fair comparison between the MSDC model and other SOTA models. Next, use the suggested hyperparameters to train and assess all models, setting the batch size to 16 and the training epochs to 20.

**Table 4:** Comparative case analysis of MSDC model and SOTA model.

Sample	SSEGCN	R-GAT	DualGCN	MSDA
Great [beer selection] too, something like 50 [beers].	P✓,P✗	P✓,P✗	P✓,N✗	P✓,O✓
As for all the fancy [finger swipes], I just gave up and attached a [mouse].	P✗,P✓	N✓,O✓	P✗,O✓	N✓,O✓
Unfortunately, it runs [XP] and Microsoft is dropping [support] next April.	N✗,N✓	N✗,N✓	N✗,N✓	O✓,N✓
The only good thing about [restaurant] is the nice [service].	P✗,P✓	N✓,P✓	O✓,P✓	N✓,P✓

In the first example, the aspect word “beer selection" contains a clear positive sentiment word “Great", which allows all models to classify it correctly. However, since “beers" does not have an obvious sentiment word, the three baseline models misclassify it. The MSDC model processes the grammatical structure at multiple scales and analyzes the different sentiment states of “beer selection" and “beers". In the second example, although “finger swipes" has an obvious sentiment clue “fancy", the term “mouse" lacks a clear sentiment word. Therefore, SSEGCN incorrectly predicts “finger swipes" and “mouse" as positive. DualGCN links “finger swipes" with “mouse" but does not consider the relationship between the grammatical structures, resulting in misclassification. In contrast, our model correctly identifies the relationship between “finger swipes" and “mouse", and accurately corresponds sentiment words to aspects through the analysis of global features. In the third example, DualGCN only connects “XP" with “support" and ignores “Unfortunately" in the sentence, resulting in “XP" being misclassified. Meanwhile, SSEGCN only focuses on the second conjunction “and” and ignores the syntactic structure, resulting in incorrect predictions. R-GAT analyzes the sentence locally, resulting in incorrect sentiment analysis of “XP”. However, our model correctly associates aspect words and sentiment through the global and local syntactic dependencies of the sentence. In the fourth example, while “service” has an obvious sentiment clue “nice”, the aspect word “restaurant” lacks a clear sentiment word. Therefore, SSEGCN incorrectly predicts “restaurant” as neutral. DualGCN associates “restaurant” with “good” but ignores the importance of the word “only” in the syntactic structure, resulting in misclassification. In contrast, our model correctly identifies the relationship between “only” and “restaurant” while reducing the impact of other noise on the experiment and accurately classifies it. In summary, the predictions that match the true labels show that the MSDC model is more effective than the baseline model in capturing sentiment and syntactic knowledge in sentences.

However, the MSDC model also exhibits certain processing limitations. These limitations can be observed in adversarial scenarios where synonym substitution (“Great" → “Good") in “Good beer selection, but the beers are average" may mislead the model to overlook the negative sentiment of “average" due to syntactic parsing errors. Complex syntactic structures like “Despite the fancy finger swipes, the mouse is not responsive" further challenge MSDC, as incorrect dissociation of “not" from “responsive" might lead to misinterpreting “mouse" as positive. Additionally, the model struggles with long-tail data such as slang terms like “YOLO" in “This restaurant’s vibe is YOLO, but the service is slow," where insufficient training on informal language could result in misclassifying “YOLO" as neutral/negative despite its inherent positivity.

Future research will address these challenges by developing adversarial training to enhance robustness, optimizing syntactic parsing for complex structures, and integrating few-shot learning to improve handling of slang and rare linguistic patterns.

## 5 Research insights and impact discussion

### 5.1 Key research questions

Extract the key research questions from the Section “4.7 Case analysis" in the experiment:

RQ1: How does multi-scale syntactic analysis resolve ambiguous sentiment scenarios without explicit opinion words? (Corresponding to Example 1: The word “beers" has no sentiment, but the MSDC associates “Great" with “beers" through syntactic dependencies).

RQ2: Why does adaptive fusion outperform static dependency modeling in handling negation cues? (Corresponding to Example 4: Capturing the negative modification of “only" to “restaurant", which is ignored by DualGCN).

RQ3: Can global syntactic context mitigate local prediction conflicts? (Corresponding to Example 3: Combining the global sentiment of “Unfortunately" to correct the local analysis of “XP").

### 5.2 Impact of the MSDC

Our work bridges two critical gaps in ABSA: (1) The proposed dual-channel architecture formally unifies syntactic dependency modeling (via GNNs) and semantic relational learning (via gated attention), whereas prior studies treated them as separate components (e.g., ASGCN uses GNN alone). As evidenced by the error reduction on contrastive clauses, this synergy enables robust cross-domain generalization. (2) The capsule-based routing protocol introduces a novel paradigm for polarity disambiguation, fundamentally differing from traditional threshold-based methods. The capsule activation patterns reveal its capability to capture sentiment intensity gradients, which may inspire new evaluation metrics beyond categorical classification. Simultaneously, in the course of future development, it holds the potential to be extended to medical text analysis, such as the implicit sentiment within patients’ descriptions of symptoms. It proffers an entirely novel paradigm for aspect-based sentiment analysis, enabling multi-scale syntactic-semantic integration, thereby augmenting the precision of sentiment analysis.

### 5.3 Threats to validity and limitations

This study has several limitations related to the dataset. First, the dataset used in this research is relatively small, which may limit the generalizability of the findings. Additionally, the dataset is imbalanced, with certain categories underrepresented, potentially biasing the model’s performance. Second, the data were collected from a specific geographic region, which may not fully capture the diversity of other contexts. Third, the dataset contains missing values and noise, which were addressed through preprocessing, but residual errors may still exist. Finally, the dataset’s temporal scope is limited to a specific period, which may not reflect current trends. Future research could address these limitations by using larger, more diverse datasets and incorporating more robust data collection methods.

## 6 Conclusion

In order to perform ABSA tasks, we developed a novel model in this study that is based on multi-scale dual-channel feature fusion (MSDC). Specifically, we utilize the neighborhood enhancement module to enrich the syntactic features’ representation, and design a multi-head gated self-attention module to extract the global attention information of aspect words, effectively capturing the semantic relevance between words; design a graph neural network module to generate rich local syntactic feature representations, highlighting the more essential neighbor nodes. The model combines the multi-head gated self-attention module with the graph neural network structure to enhance the syntactic information features, capture high-dimensional features from a global and local perspective, and thus solve the semantic matching problem existing in traditional syntactic dependency analysis. Then, the adaptive feature fusion module is used to fuse multi-scale sentiment features to comprehensively capture and understand the text’s subtle sentiment and semantic differences. Finally, in order to provide a more refined feature representation, the capsule network module is utilized to further integrate and process the fine-grained feature information while lowering the noise impact in the ABSA tasks.

Through the planned comparison and ablation tests, the efficacy of the network model put out in this research is confirmed. ABSA has now produced some outcomes in the dual-channel fusion sector. In future work, based on this research, we will combine data and knowledge from more fields to conduct ABSA to improve the generalization and adaptability of the model.

## Supporting information

S1 Text MSDC. (PDF)
