# Supplementary material for: MSDC: Aspect-level sentiment analysis model based on multi-scale dual-channel feature fusion
Source: PLoS One. 2025 Oct 21;20(10):e0328839. doi: 10.1371/journal.pone.0328839 (PMC12539747; doi:10.1371/journal.pone.0328839)
Supplement: S1 Text — (PDF) [file pone.0328839.s001.pdf]

```
from transformers.models.bert.modeling_bert import BertPooler, BertSelfAttention
import torch
import torch.nn as nn
import torch.nn.functional as F
import numpy as np
from transformers import BertConfig
class GraphConvolution(nn.Module):
    def __init__(self, in_features, out_features, bias=True):
        super(GraphConvolution, self).__init__()
        self.in_features = in_features
        self.out_features = out_features
        self.weight = nn.Parameter(torch.FloatTensor(in_features, out_features))
        if bias:
            self.bias = nn.Parameter(torch.FloatTensor(out_features))
        else:
            self.register_parameter('bias', None)


    def forward(self, text, adj):
        hidden = torch.matmul(text, self.weight)
        denom = torch.sum(adj, dim=2, keepdim=True) + 1
        output = torch.matmul(adj, hidden) / denom
        if self.bias is not None:
            return output + self.bias
        else:
            return output
class GraphAttentionLayer(nn.Module):
    def __init__(self, in_features, out_features, dropout, alpha, concat=True):
        super(GraphAttentionLayer, self).__init__()
        self.in_features = in_features
        self.out_features = out_features
        self.dropout = dropout
        self.alpha = alpha
        self.concat = concat
        self.W = nn.Parameter(torch.zeros(size=(in_features, out_features)))
        nn.init.xavier_uniform_(self.W.data, gain=1.414)
        self.a = nn.Parameter(torch.zeros(size=(2*out_features, 1)))
        nn.init.xavier_uniform_(self.a.data, gain=1.414)
```

```
        self.leakyrelu = nn.LeakyReLU(self.alpha)
    def forward(self, h, adj):
        Wh = torch.matmul(h, self.W)
        e = self._prepare_attentional_mechanism_input(Wh)
        zero_vec = -9e15*torch.ones_like(e)
        attention = torch.where(adj > 0, e, zero_vec)
        attention = F.softmax(attention, dim=1)
        attention = F.dropout(attention, self.dropout, training=self.training)
        h_prime = torch.matmul(attention, Wh)
        if self.concat:
            return F.elu(h_prime)
        else:
            return h_prime
    def _prepare_attentional_mechanism_input(self, Wh):
        B = Wh.shape[0]
        seq_len = Wh.shape[1]

        Wh1 = Wh.unsqueeze(2).expand(B, seq_len, seq_len, -1)
        Wh2 = Wh.unsqueeze(1).expand(B, seq_len, seq_len, -1)

        e = torch.cat([Wh1, Wh2], dim=-1)
        e = torch.matmul(e, self.a).squeeze(-1)
        return self.leakyrelu(e)
class EnhancedGraphConvolution(nn.Module):

    def __init__(self, in_features, out_features, dropout=0.6, alpha=0.2, bias=True):
        super(EnhancedGraphConvolution, self).__init__()
        self.gcn = GraphConvolution(in_features, out_features, bias=bias)
        self.gat = GraphAttentionLayer(out_features, out_features, dropout, alpha,
concat=False)
    def forward(self, text, adj):
        gcn_out = self.gat(text,adj)
        gcn_out = self.gcn(gcn_out, adj)
        gcn_out = self.gcn(gcn_out, adj)
        return gcn_out
class CapsuleLayer(nn.Module):
    def __init__(self, num_capsules, num_route_nodes, in_channels, out_channels,
```

```
kernel_size=None, stride=None, num_iterations=3):
        super(CapsuleLayer, self).__init__()
        self.num_route_nodes = num_route_nodes
        self.num_capsules = num_capsules
        self.num_iterations = num_iterations

        if num_route_nodes != -1:
            self.route_weights = nn.Parameter(torch.randn(num_capsules,
num_route_nodes, in_channels, out_channels))
        else:
            self.capsules = nn.ModuleList(
                [nn.Conv2d(in_channels, out_channels, kernel_size=kernel_size,
stride=stride, padding=0) for _ in range(num_capsules)]
            )

    def squash(self, tensor, dim=-1):
        squared_norm = (tensor ** 2).sum(dim=dim, keepdim=True)
        scale = squared_norm / (1 + squared_norm)
        return scale * tensor / torch.sqrt(squared_norm + 1e-8)

    def forward(self, x):
        if self.num_route_nodes != -1:
            priors = x[None, :, :, None, :] @ self.route_weights[:, None, :, :, :]
            logits = torch.zeros(*priors.size()).to(x.device)
            for i in range(self.num_iterations):
                probs = F.softmax(logits, dim=2)
                outputs = self.squash((probs * priors).sum(dim=2,
keepdim=True))
                if i != self.num_iterations - 1:
                    delta_logits = (priors * outputs).sum(dim=-1, keepdim=True)
                    logits = logits + delta_logits
            return outputs.squeeze(3)
        else:
            outputs = [capsule(x).view(x.size(0), -1, 1) for capsule in self.capsules]
            outputs = torch.cat(outputs, dim=-1)
            return self.squash(outputs)
```

```
class SelfAttention(nn.Module):
    def __init__(self, config, opt):
        super(SelfAttention, self).__init__()
        self.opt = opt
        self.config = config

        self.SA = BertSelfAttention(config)

        self.layer_norm1 = nn.LayerNorm(config.hidden_size)
        self.layer_norm2 = nn.LayerNorm(config.hidden_size)

        self.feed_forward = nn.Sequential(
            nn.Linear(config.hidden_size, config.intermediate_size),
            nn.ReLU(),
            nn.Linear(config.intermediate_size, config.hidden_size)
        )

        self.gate_linear = nn.Linear(2 * config.hidden_size, config.hidden_size)
        self.sigmoid = nn.Sigmoid()
        self.tanh = nn.Tanh()

    def forward(self, inputs):

        zero_tensor = torch.zeros((inputs.size(0), 1, 1, self.opt.max_seq_len),
dtype=torch.float32).to(self.opt.device)

        attention_output = self.SA(inputs, zero_tensor)
        attention_output = attention_output[0]

        attention_output = self.layer_norm1(inputs + attention_output)

        feed_forward_output = self.feed_forward(attention_output)

        feed_forward_output = self.layer_norm2(attention_output +
feed_forward_output)

        concat_output = torch.cat((attention_output, feed_forward_output), dim=-1)
```

```
            gate = self.sigmoid(self.gate_linear(concat_output))
            gated_output = gate * self.tanh(feed_forward_output) + (1 - gate) *
attention_output

            return gated_output
class MSDC(nn.Module):
      def __init__(self, bert, opt):
            super(MSDC, self).__init__()
            self.bert_spc = bert
            self.opt = opt
            self.dropout = nn.Dropout(opt.dropout)
            self.bert_SA = SelfAttention(bert.config, opt)
            self.linear_double = nn.Linear(opt.bert_dim * 2, opt.bert_dim)
            self.linear_single = nn.Linear(opt.bert_dim, opt.bert_dim)
            self.bert_pooler = BertPooler(bert.config)
            self.dense = nn.Linear(opt.bert_dim, opt.polarities_dim)
            self.pool0 = nn.AdaptiveAvgPool2d(output_size=(1, 1))
            self.pool = nn.AvgPool1d(opt.threshold + 1)
            self.enhance = EnhancedGraphConvolution(768,768)
            self.gcn = GraphConvolution(768, 768, bias=True)
            self.capsule_layer = CapsuleLayer(
                  num_capsules=10,    # e.g., 10
                  num_route_nodes=-1,
                  in_channels=opt.bert_dim,    # Input channels
                  out_channels=16,    # Output dimension of each capsule, e.g., 16
                  kernel_size=1,    # Kernel size for Conv2d capsules
                  stride=1,
                  num_iterations=3
            )
            self.dense_caps = nn.Linear(1600*10, opt.polarities_dim)

      def moving_mask(self, text_local_indices, aspect_indices, mask_len):
            texts = text_local_indices.cpu().detach().numpy()
            asps = aspect_indices.cpu().numpy()
            masked_text_raw_indices = np.ones((text_local_indices.size(0),
self.opt.max_seq_len, self.opt.bert_dim),
                                                                  dtype=np.float32)
            for text_i, asp_i in zip(range(len(texts)), range(len(asps))):
```

```
                asp_len = np.count_nonzero(asps[asp_i]) - 2
                try:
                    asp_begin = np.argwhere(texts[text_i] == asps[asp_i][1])[0][0]
                except:
                    continue
                if asp_begin >= mask_len:
                    mask_begin = asp_begin - mask_len
                else:
                    mask_begin = 0
                for i in range(mask_begin):
                    masked_text_raw_indices[text_i][i] =
np.zeros((self.opt.bert_dim), dtype=np.float)
                for j in range(asp_begin + asp_len + mask_len, self.opt.max_seq_len):
                    masked_text_raw_indices[text_i][j] =
np.zeros((self.opt.bert_dim), dtype=np.float)
        masked_text_raw_indices = torch.from_numpy(masked_text_raw_indices)
        return masked_text_raw_indices.to(self.opt.device)

    def position_weight(self, x, aspect_double_idx, text_len, aspect_len):
        batch_size = x.shape[0]
        seq_len = x.shape[1]
        aspect_double_idx = aspect_double_idx.cpu().numpy()
        text_len = text_len.cpu().numpy()
        aspect_len = aspect_len.cpu().numpy()
        weight = [[] for i in range(batch_size)]
        for i in range(batch_size):
            context_len = text_len[i] - aspect_len[i]
            for j in range(aspect_double_idx[i, 0]):
                weight[i].append(1 - (aspect_double_idx[i, 0] - j) / context_len)
            for j in range(aspect_double_idx[i, 0], aspect_double_idx[i, 1] + 1):
                weight[i].append(0)
            for j in range(aspect_double_idx[i, 1] + 1, text_len[i]):
                weight[i].append(1 - (j - aspect_double_idx[i, 1]) / context_len)
            for j in range(text_len[i], seq_len):
                weight[i].append(0)
        weight = torch.tensor(weight,
dtype=torch.float).unsqueeze(2).to(self.opt.device)
        return weight * x
```

```
    def forward(self, inputs):
        text_bert_indices = inputs[0]
        bert_segments_ids = inputs[1]
        text_local_indices = inputs[2]
        aspect_indices = inputs[3]
        adj = inputs[4]
        left_indices = inputs[5]

        text_len = torch.sum(text_bert_indices != 0, dim=-1)
        aspect_len = torch.sum(aspect_indices != 0, dim=-1)
        left_len = torch.sum(left_indices != 0, dim=-1)
        aspect_double_idx = torch.cat([left_len.unsqueeze(1), (left_len + aspect_len - 1).unsqueeze(1)], dim=1)

        bert_spc_out, _ = self.bert_spc(text_bert_indices, token_type_ids=bert_segments_ids, return_dict=False)
        bert_spc_out = self.dropout(bert_spc_out)

        neighboring_span, _ = self.bert_spc(text_local_indices, return_dict=False)
        neighboring_span = self.dropout(neighboring_span)

        out_list = []
        for i in range(self.opt.threshold + 1):

            masked_local_text_vec = self.moving_mask(bert_spc_out, aspect_indices, i)
            neighboring_span = torch.mul(neighboring_span, masked_local_text_vec)
            enhanced_text = torch.cat((neighboring_span,bert_spc_out), dim=-1)
            enhanced_text = self.dropout(enhanced_text)
            mean_pool = self.linear_double(enhanced_text)
            x = torch.relu(mean_pool)
            seq_len = x.shape[1]
            adj = adj[:, :seq_len, :seq_len]
            y = self.position_weight(x, aspect_double_idx, text_len, aspect_len)
            y1 = F.relu(self.enhance(y, adj))
            y2 = self.pool0(y1)
```

```
            y3 = torch.sigmoid(y2)
            z = self.bert_SA(x)
            z1 = self.pool0(z)
            out = AFFM(z1,y3)
            capsule_input = out.permute(0, 2, 1).unsqueeze(-1)    # Shape:
(batch_size, in_channels, seq_len, 1)
            capsule_output = self.capsule_layer(capsule_input)    # Shape: depends
on CapsuleLayer configuration
            capsule_output = capsule_output.view(capsule_output.size(0), -1)
            dense_out = self.dense_caps(capsule_output)
            out_list.append(dense_out)
        out = torch.cat(out_list, dim=-1)
        out = out.view(dense_out.shape[0], 3, -1)
        ensem_out = self.pool(out)
        ensem_out = ensem_out.squeeze(-1)
        return ensem_out
```
